# Tremor Types in Parkinson Disease: A Descriptive Study Using a New Classification

**DOI:** 10.1155/2018/4327597

**Published:** 2018-09-30

**Authors:** Alexandre Gironell, Berta Pascual-Sedano, Ignacio Aracil, Juan Marín-Lahoz, Javier Pagonabarraga, Jaime Kulisevsky

**Affiliations:** Movement Disorders Unit, Department of Neurology, Hospital de la Santa Creu i Sant Pau, Autonomous University of Barcelona, Catalonia, Spain

## Abstract

**Background:**

The current classification of tremor types in Parkinson disease (PD) is potentially confusing, particularly for mixed tremor, and there is no label for pure resting tremor. With a view to better defining the clinical phenomenological classification of these tremors, our group relabeled the different types as follows: pure resting tremor (type I); mixed resting and action tremor with similar frequencies (type II) divided, according to action tremor presentation, into II-R when there is a time lag and II-C otherwise; pure action tremor (type III); and mixed resting and action tremor with differing frequencies (type IV). We performed a descriptive study to determine prevalence and clinical correlates for this new tremor classification.

**Patient/Methods:**

A total of 315 consecutively recruited patients with PD and tremor were clinically evaluated. *X*^2^ tests were used to assess tremor type associations with categorical variables, namely, sex, family history of PD, motor fluctuations, and anticholinergic and beta-blocker use. With tremor type as the independent variable, ANOVA was performed to study the relationship between dependent quantitative variables, namely, age, age at PD diagnosis, disease duration, and UPDRS scores for rigidity.

**Results:**

The studied patients had tremor types as follows: type I, 30%; type II, 50% (II-R, 25% and II-C, 25%); type III, 19%; and type IV, 1%. No significant association was found between the studied clinical variables and tremor types.

**Conclusions:**

Mixed tremor was the most common tremor type in our series of patients with PD according to our proposed classification, which we hope will enhance understanding of the broad clinical phenomenology of PD.

## 1. Introduction

Tremor, one of the most characteristic manifestations of Parkinson disease (PD) and often the presenting sign, occurs in approximately 75% of patients with PD, who rank it as their second most troublesome symptom [1, 2].

PD tremor, whether resting or action tremor, is a highly variable symptom [3]. The classical tremor is reported as a resting tremor of 4–7 Hz, often asymmetric, which ceases with volitional movement [4]. Only resting tremor is a positive diagnostic criterion for PD [4, 5]. However, action tremor, whether pure or mixed with resting tremor, is often observed in PD and has a reported prevalence of as high as 92% [5].

In 1998, the Consensus Statement on Tremor issued by the Movement Disorder Society (MDS) classified the different tremor types observed in PD in 3 categories: type I, resting and action tremor with similar frequencies; type II, resting and action tremor with differing frequencies; and type III, pure action tremor. Finally, the term monosymptomatic rest tremor refers to patients with rest tremor and no other parkinsonian signs [6]. A more recent revised Consensus Statement does not modify the tremor type classification for PD [7]. In our opinion, this classification has led to some confusion regarding mixed tremor. In particular, differing tremor frequencies in type II tremor suggest that different tremor pacemakers are used and also possibly indicate the coexistence of different diseases. Furthermore, pure resting tremor does not appear as a separate tremor type.

With a view to better defining the clinical phenomenological classification of PD, our group relabeled the different PD tremor types in what appeared to us to be a more intuitive and logical way, as follows: type I, pure resting tremor; type II, mixed action and resting tremor with similar frequencies (subdivided into re-emergent and continuous tremors); type III, pure action tremor; and type IV, mixed action and resting tremor with different frequencies.

Below we describe a descriptive study of prevalence and clinical correlates for this new tremor classification, based purely on clinical phenomenology, for a consecutive series of patients with PD.

## 2. Patients and Methods

Included consecutively in the study were patients with PD and visibly detected tremor who attended the Movement Disorders Unit at Sant Pau Hospital Neurology Department between December 2015 and April 2017 (16 months). PD was diagnosed using the London Brain Bank criteria [8]. The study protocol was approved by the hospital ethics committee, and the study was performed in accordance with international ethical regulations. All subjects granted their signed informed consent to participate. The patients were clinically evaluated by neurologists specializing in movement disorders [9, 10].

Resting tremor was defined as a tremor present when arms were resting in the lap or by the side. Since it was not always easy to ensure that a patient was completely relaxed, when in doubt and in order to rule out gravitational force and muscle co-contraction, the patient was tested lying down on a bed or walking with their arms hanging at the sides. We also asked patients to complete tasks that increased tremor amplitude, either mental tasks (e.g., saying the months of the year backwards) or motor tasks (e.g., opening and closing the contralateral hand).

Action tremor can be either kinetic or postural. Kinetic tremor was defined as tremor present during hand movement (e.g., during a finger-to-nose movement or when writing). Postural tremor was assessed for stretched-out arms, the bat-wing position, and wrist extension.

For mixed tremor, accelerometric recordings were made using a previously described methodology [11]. Resting and action tremor were considered to have similar frequencies when the difference in frequency peaks was below 1.5 Hz. This is the described cutoff value for the coexistence of different tremor pacemakers and syndromes [12]. The clinical evaluation was performed in OFF time (12 hours after the last levodopa dose).

**Figure 1:**
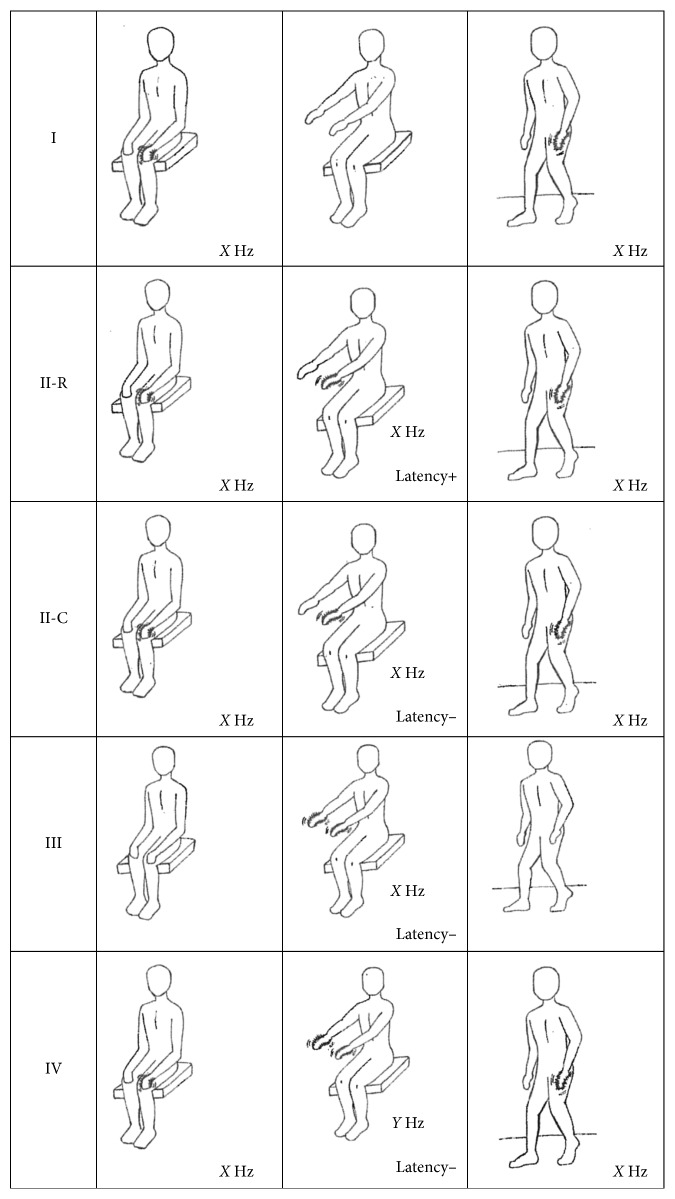
Tremor types in Parkinson disease graphically explained. Difference in X and Y frequency is above 1.5 Hz.

The tremor types are summarized in Table 1 and graphically explained in Figure 1. Type I is pure resting tremor, including the classical pill-rolling tremor. Type II is mixed resting and action tremor with similar frequencies, divided, according to action tremor presentation, into type II-R (re-emergent) when there is a time lag (usually 5–20 seconds) and type II-C (continuous) when there is no time lag. Type III is pure action tremor (essential tremor-like). Finally, type IV represents mixed resting and action tremor with differing frequencies, possibly indicating the coexistence of two tremor conditions (e.g., essential tremor and PD).

In the neurological examination, for the most affected arm in each patient, the rigidity item from the motor evaluation section of the Unified Parkinson Disease Rating Scale (UPDRS) was used as a measure of parkinsonism severity. The Hoehn and Yahr scale for describing progression of PD symptoms was also scored. Also assessed based on a chart review and on patient reports were several clinical factors correlating with PD tremor types: age, sex, family history of tremor, disease duration, motor fluctuations, and treatment with anticholinergics or beta-blockers.

For the descriptive analysis, categorical variables were reported as percentages and number of cases, and quantitative variables were reported as mean ± standard deviation. *X*^2^ tests were used to assess tremor type associations with the categorical variables of sex, family history of PD, presence of motor fluctuations, anticholinergic use, and beta-blocker use. Analysis of variance (ANOVA) was performed to study the relationship between dependent quantitative variables—age, age at PD diagnosis, disease duration, and UPDRS scores for rigidity—with tremor type as the independent variable. For all analyses, the statistical level of significance was established at 5% (alpha = 0.05, two-sided). The statistical package used was IBM-SPSS (ver. 22.0).

## 3. Results

A total of 315 patients (166 men and 149 women) with a mean age of 76.4 years (range 60–85 years) were included in the study.  Table 2 shows the demographic and clinical characteristics of the patients in our series.  Table 3 shows the accelerometric data for mixed tremor.

Around 30% of patients had tremor type I, 50% had type II (25% II-R and 25% II-C), 19% had type III, and 1% had type IV.

No significant association was found between the clinical variables studied and tremor types: age (*p*=0.232), sex (*p*=0.092), disease duration (*p*=0.238), Hoehn and Yahr score (*p*=0.358), family history of PD (*p*=0.766), percentage of motor fluctuations (*p*=0.788), and rigidity score (*p*=0.990). There were no statistically significant differences in the percentage of patients with each tremor type in terms of anticholinergic use. No patient with type I tremor in our series was taking beta-blockers.

## 4. Discussion

We propose new labels for tremor types observed in PD based purely on clinical phenomenology. In our series, we found the most common tremor presentation in PD to be mixed resting and action tremor (51% of the patients). In our opinion, this new classification improves on the MDS classification as it is more intuitive and logical. Our classification based on a descriptive cross-sectional study does not have a pathological, prognostic, or therapeutic value, but represents a first step for future pathological and prospective studies to determine its usefulness.

There are two main differences between our classification and the MDS classification [6, 7]. We include pure resting tremor (type I), and we emphasize mixed tremor with its respective categories, i.e., the same frequency (type II-R or II-C) or a different frequency (type IV).

Our study found that action tremor is prevalent in PD. In fact, pure or mixed action tremor was experienced by 70% of the patients in our series, a finding that corroborates previous studies reporting action tremor prevalence of as high as 92% in patients with PD [4, 5, 13, 14]. Nonetheless, only resting tremor is a diagnostic criterion for PD [15]. Yet pure resting tremor was experienced by only 30% of the patients in our study, whereas resting tremor including mixed tremor was experienced by 81% of our patients.

Interestingly, we found that 19% of our patients had pure action tremor, that is, essential tremor-like tremor. Our group has previously reported that this may be the first symptom of PD in about 6% of cases [16]. While some consider this tremor to be of minor importance, in clinical practice, moderate action tremor is regarded as more disabling than severe resting tremor [4, 5, 13, 14, 17].

The basis for action tremor in PD is not clear [5]. Several studies suggest that action tremor, like resting tremor, may be a manifestation of underlying basal ganglia disease [18]. Tremor has been reported to be more pronounced on the side of the body that is most affected by PD [19]. Single-dose challenges of dopaminergic and anticholinergic agents have been reported to significantly reduce action tremor amplitude [20]. Furthermore, a significant correlation between action tremor and resting tremor has been reported, suggesting that action tremor is a variant of resting tremor in patients with PD [21]. In a recent study, Dirkx et al. suggest that there are two distinct postural tremor phenotypes in PD: re-emergent tremor as a continuation of resting tremor during stable posturing, with a dopaminergic basis, and pure postural tremor as a less common type of tremor in PD, but with a largely nondopaminergic basis [22].

It has been hypothesized that re-emergent tremor (type II-R in our classification) is a form of resting tremor in PD [23]. Few studies have distinguished between re-emergent tremor and tremor that does not occur after a time lag [24]. In our study, we found similar prevalence for both re-emergent and continuous mixed tremor. Interestingly, a recent study reported a significantly shorter illness duration for patients with re-emergent tremor [25]. In our study, however, we found no difference in disease duration for the different tremor types. Furthermore, we found no relationship between tremor type and other clinical manifestations of PD, including rigidity, Hoehn and Yahr score, or PD family history.

The main limitation of our study is the small number of patients included, although we suggest that the sample is quite representative. Furthermore, we did not control for the effects of antiparkinsonian medication that might modify the tremor phenomenology. However, the patients in our study were evaluated in OFF time.

Furthermore, as a weighted limb test was not performed, some degree of misdiagnosis cannot be ruled out in cases of physiological action tremor. The classification of pure action tremor is difficult because rest tremor may be very mild and intermittent, especially when patients are treated with dopaminergic drugs.

Finally, another limitation of this cross-sectional study is that we did not study shifts between tremor subtypes; i.e., we did not assess how many patients switched from one type of tremor to another, although the absence of any significant correlation between disease duration and tremor type would suggest that shifting is not relevant. This issue, however, would need to be analyzed in future studies.

## 5. Conclusion

We propose new labels for tremor types observed in PD based purely on clinical phenomenology. Mixed tremor was the most common tremor type in our series of patients with PD according to our proposed classification, which we hope will enhance understanding of the broad clinical phenomenology of PD.

## Figures and Tables

**Table 1:** Tremor types in Parkinson disease.

**Type I**. Pure resting tremor
**Type II**. Mixed resting and action tremor with similar frequencies^*∗*^
** II-R**. Action tremor with a time lag
** II-C**. Action tremor without a time lag
**Type III**. Pure action tremor
**Type IV**. Mixed resting and action tremor with different frequencies^*∗∗*^

^*∗*^Difference <1.5 Hz; ^*∗∗*^difference >1.5 Hz.

**Table 2:** Characteristics of patients with Parkinson disease by tremor type.

Characteristics	Tremor type					Association
	I	II-R	II-C	III	IV	
Number	96	78	78	60	3	—
Age	76.2 ± 9.2	76.2 ± 9.6	72.7 ± 9.1	74.0 ± 9.5	83.1 ± 1.4	*p*=0.232
Female (%)	57	53	53	41	60	*p*=0.092
Family history of PD (%)	5	10	17	16	20	*p*=0.766
Disease duration (years)	5.2 ± 3.1	5.1 ± 3.2	7.3 ± 6.0	6.4 ± 4.7	5.5 ± 3.5	*p*=0.238
Hoehn and Yahr I-II (%)	92	95	90	90	85	*p*=0.358
Motor fluctuations (%)	12	10	12	9	10	*p*=0.788
UPDRS rigidity	2.1 ± 0.3	2.0 ± 0.23	2.0 ± 0.3	2.2 ± 0.1	2.0 ± 0.3	*p*=0.990
Anticholinergics (%)	15	25	24	10	10	*p*=0.110
Beta-blockers (%)	0	10	30	50	60	*p*=0.090

UPDRS, Unified Parkinson's Disease Rating Scale. Association: statistical association between clinical variable and tremor type.

**Table 3:** Accelerometric data for patients with mixed tremor.

Tremor type	Resting tremor (Hz)	Action tremor (Hz)	Time lag (seconds)
II-R	5.0 ± 0.8	4.9 ± 1.1	6.8 ± 2.2
II-C	5.2 ± 0.9	5.1 ± 0.7	0
IV	4.8 ± 1.1	7.5 ± 0.9	0

## Data Availability

The data used to support the findings of this study are available from the corresponding author upon request.
